# Polyethylene Transformation Chain: Evaluation of Migratable Compounds

**DOI:** 10.3390/polym17030295

**Published:** 2025-01-23

**Authors:** Patricia Vázquez-Loureiro, Nuria García-Batista, Antonio Morreale, Raquel Llorens-Chiralt, Hernando Villar, Beatriz Bacaicoa, Ana Rodríguez Bernaldo de Quirós, Raquel Sendón

**Affiliations:** 1 Department of Analytical Chemistry, Nutrition and Food Science, Faculty of Pharmacy, University of Santiago de Compostela, 15782 Santiago de Compostela, Spain; patriciavazquez.loureiro@usc.es (P.V.-L.); ana.rodriguez.bernaldo@usc.es (A.R.B.d.Q.); 2 Instituto de Materiales (iMATUS), University of Santiago de Compostela, 15782 Santiago de Compostela, Spain; 3 AIMPLAS, Plastics Technology Centre, Gustave Eiffel, 4, 46980 Paterna, Spain; ngarcia@certest.es (N.G.-B.); rllorens@aimplas.es (R.L.-C.); 4 Repsol Química, S.A., Agustín de Betancourt, s/n, 28935 Móstoles, Spain; antonio.morreale@repsol.com; 5 Bacaicoa, Carretera Pamplona-Irún, km. 6,7, 31194 Oricain, Spain; hvillar@bacaicoaip.es (H.V.); beatriz@bacaicoaip.es (B.B.)

**Keywords:** polyethylene, PE films, GC-MS, P&T-GC-MS, NIAS

## Abstract

Polyethylene (PE) is a widely used material for packaging food. However, certain additives and their degradation products, which may be generated during transformation processes, may pose risks to consumers health if they migrate into food at levels exceeding safety thresholds. Therefore, identifying and quantifying these potential migrant compounds is crucial to ensuring consumer safety. In the present work, PE films and the raw materials used in their production were kindly provided by the industry to evaluate undesired compounds throughout the PE transformation chain. For that purpose, volatile and semi-volatile organic compounds were evaluated using gas chromatography coupled to mass spectrometry (GC-MS). Alkanes were identified as the most abundant compounds, along with antioxidants, lubricants, or Non-Intentionally Added Substances (NIAS), like 7,9-di-tert-butyl-1-oxaspiro(4,5)deca-6,9-diene-2,8-dione in the films. For the unidentified compounds, evaluations were conducted at various stages of the transformation chain, and migration assays were performed to assess their behavior.

## 1. Introduction

Food-contact materials (FCMs) can be manufactured from a wide variety of materials, and they can contain Non-Intentionally Added Substances (NIAS) because of the interactions between different ingredients in the packaging materials, such as degradation processes [1] and impurities present in the raw materials used in the manufacturing process [2]. In this sense, the regulation on plastic FCMs (Regulation 10/2011/EU) [3] recognizes that NIAS can be formed during the manufacture and use of plastic materials and articles. In addition, according to this regulation, any potential health risks arising from exposure to NIAS should be assessed by the manufacturer in accordance with internationally recognized scientific principles of risk assessment.

Most food plastic packages are based on polyolefins, such as polyethylene (PE) or polypropylene (PP), which are saturated polymers that can be degraded when exposed to ultraviolet radiation from sunlight or oxidized at high temperatures. To protect the polymer during its processing, additives are included in the formulation of these materials [4], and these low molecular weight compounds have also been introduced to improve the physical and chemical properties of packaging [5,6]. However, it has been reported that these compounds can also produce degradation products. For example, the exposure of food packaging to microwave heating can cause a higher degradation of the antioxidants Irgafos 168 and Irganox 1010 [7] to yield 2,4-di-tert-butylphenol (2,4-DTBP), 2,6-di-tert-butyl-p-benzoquinone (2,6-DTBQ), 3,5-di-tert-butyl-4-hydroxyphenylpropionic acid, 2,6-di-tert-butyl-4-methoxyphenol, 3,5-di-tert-butyl-4-hydroxybenzoic acid, triphenyl phosphate, tri-o-tolyl phosphate, diphenyl phosphate, and 3-(3,5-di-tert-butyl-4-hydroxybenzyl) propionic acid. Moreover, other degradation products, such as alkylphenols, have also been reported because of their toxicity, as they are endocrine disrupters [8].

In this sense, PE-based packaging materials undergo several intermediate treatments during their production, such as the application of adhesives to bond multiple layers and the use of printing inks. It is nearly impossible to determine in detail which specific compounds are present in the final packaging material. Furthermore, the polymers and additives used can degrade during processing, leading to the formation of additional contaminants [9]. Identifying these undesired substances in the final product is a significant challenge, since not only can these compounds be present, but also other endocrine disruptors [10], esters, aldehydes, alcohols, ketones, hydrocarbons, etc. This last family of compounds is usually the main volatile one identified in plastics, and its content is even higher in recycled plastic than in virgin PE, showing that the content of NIAS could be affected by the recycling process of plastics [11,12].

The safety of PE when used as an FCM is reflected in numerous published works [1,2,7,9]. However, the objective of this study was to evaluate and reduce NIAS along the transformation chain from raw materials to films (final FCMs). This approach provides an evaluation of NIAS that could potentially migrate to the food, thereby enhancing consumer health protection.

In this work, a non-targeted analysis was conducted for the identification of unknown compounds in different grades of polyethylene, as well as in the additives and raw materials used in their manufacture and during their transformation. This study is very ambitious, as it includes the analysis of material at different stages of film production with the aim of identifying chemical substances prone to migration, thus evaluating how to reduce their formation. It comprises samples from raw materials to final products intended to be in contact with food and evaluates the intermediate compounding stage. For this purpose, volatile compounds were determined by gas chromatography and mass spectrometry coupled to purge and trap (P&T-GC-MS) in different samples collected at different stages of the manufacturing process, whereas semi-volatile compounds were determined in samples by gas chromatography coupled to mass spectrometry (GC-MS) after solvent extraction.

## 2. Materials and Methods

### 2.1. Sample Description

**Figure 1:**
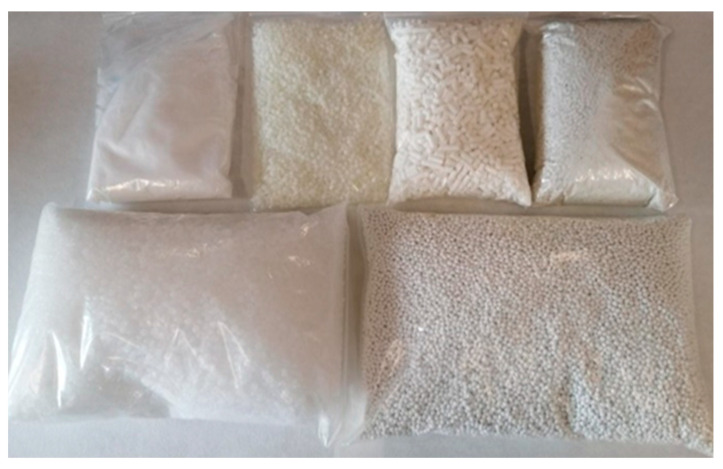
Additives for PE (Add.1, Add.2, Add.3 and Add.4 on the top, from left to right, respectively and Add.5 and Add.6 below, from left to right respectively.

Samples of three different grades of polyethylene in pellet form were kindly supplied by one company that participated in this research, namely Repsol Química, S.A., Móstoles, Spain. Likewise, additives and processing aids were analyzed, as well as the films obtained as the final product, which will be in contact with food. All samples are described in Table 1. The appearance of the additives is shown in Figure 1.

Furthermore, the above-mentioned PE grades were extruded using a ZSK25 extruder (Coperion GmbH, Stuttgart, Germany) employing a temperature range from 204 to 242 °C and under the different conditions detailed in Table 2. This was conducted to investigate the formation of potential degradation products influenced by these conditions.

The films manufactured with these materials are described in Table 3. Nine films were produced by modifying specific parameters while maintaining the same structure. Each film consisted of three layers: the outer layers were 12.5 µm thick, while the core layer was 25 µm thick. The composition of the core layer, which affects the film’s final appearance (transparent or white-opaque), was modified as shown in Table 3.

After comparing the results obtained with the different processing methods for PE2 (which is a metallocene like PE3) and considering that the emission of volatiles is much lower for PE3 than for any improvement made in PE2, a PE3 manufactured with the conditions pre-established by the manufacturer was used.

### 2.2. Sample Analysis

#### 2.2.1. Volatile Compounds

The volatile compounds were directly analyzed using the P&T-GC-MS system described below. For this purpose, approximately 1 g of each sample was accurately weighed into a 40 mL vial and an aliquot of the internal standard solution (prepared as described in Section 2.3) was added before the vial was hermetically sealed.

#### 2.2.2. Semi-Volatile Compound Extraction

To identify the semi-volatile compounds, a previous step of sample extraction was needed. Two different procedures were applied to approximately 800 mg of each raw material (powder and pellet). In one case, the sample was extracted with 10 mL of a mixture of hexane: ethanol (3:1) at 20 °C for 8 h (E1); in the other, 10 mL of hexane at 70 °C for 4 h (E2). In both cases, 1 mL was filtered with a 0.22 µm PTFE filter, adding at this moment the internal standard and injected in the GC-MS.

Extraction E2 was applied exclusively to the films, as it was considered the worst-case scenario. The extraction was carried out only on the food contact side using migration cells. Approximately 400 mg of the sample was weighed and extracted with 30 mL of hexane at 70 °C for 4 h. A concentration step was necessary to analyze these samples: 10 mL of each extract was concentrated with nitrogen gas until dry and redissolved with 1 mL of hexane containing the internal standard. Then, it was filtered with a 0.22 µm PTFE filter and injected in the GC-MS.

#### 2.2.3. Migration Tests

Different food simulants were assayed in the migration tests as specified in Regulation 10/2011/EU for plastic materials in contact with food. The following food simulants were used: A (ethanol 10% *v*/*v*), B (acetic acid 3% *v*/*v*), and ethanol 95% *v*/*v* (as D2 simulant substitute). Migration tests were conducted for 10 days at 40 °C. Extracts were concentrated 10 times through nitrogen steam to dryness, and the extracts were redissolved in methanol containing the internal standard. Then, it was filtered with a 0.22 µm PTFE filter and injected in the GC-MS.

### 2.3. Standards

Toluene (CAS, 108-88-3) for analysis, with 99.5% purity; acetic acid (CAS, 64-19-7), with 100% purity; ethanol (CAS, 64-17-5) for analysis; methanol, gas chromatography MS grade were supplied by Merck (Darmstadt, Germany); hexanal (CAS, 66-25-1); α-pinene (CAS, 80-56-8), with 98% purity; 2,6-di-tert-butyl-p-benzoquinone (CAS, 719-22-2), with 98% purity; 2,4-di-tert-butylphenol (CAS, 96-76-4) with 99% purity; erucamide (CAS, 112-84-5), with ˃85% purity; methyl hexadecanoate (CAS, 112-39-0), with 97% purity; tris(2,4-di-tert-butylphenyl) phosphite (Irgafos 168) (CAS, 31570-04-4) with 98% purity; and pentaerythritol tetrakis(3,5-di-tert-butyl-4-hydroxyhydrocinnamate (Irganox 1010) (CAS, 6683-19-8) with 98% purity were supplied by Sigma Aldrich (Steinheim, Germany). C7-C30 saturated alkane standard was supplied by Supelco.

The internal standards were toluene-d8 (CAS, 2037-26-5) with 99.6% purity and diethyl phthalate-3,4,5,6-d4 (DEP-d4) (CAS, 93952-12-6) with 99.3% f purity. Both were supplied by Sigma Aldrich (Steinheim, Germany).

The solution of internal standard with a concentration of 1000 mg/L was prepared in methanol. For the analysis of volatile compounds, intermediate solutions of 10 mg/L and 1 mg/L of toluene d-8 were prepared and 100 µL of solution 1 mg/L was added to the samples. For the analysis of semi-volatile compounds, 50 µL of a solution of DEP-d4 in hexane of 100 mg/L was added to the sample extracts (5 mg/L).

### 2.4. Gas Chromatography Conditions

#### 2.4.1. P&T-GC-MS

For the analysis of potential volatile compounds emitted by all the samples, a previous step of concentration was performed using an Atomx XYZ Purge and Trap (P&T) system (Teledyne LABS, Mason, OH, USA) controlled with the Atomx XYZ TekLink software version 1.2.7416.21009. The experimental conditions of the P&T were as follows: Vocarb^TM^ 3000 trap, sample temperature of 60 °C, helium as the purge gas at 40 mL/min, purge time of 30 min, desorption time of 2 min, desorption temperature of 250 °C, and desorb flow of 400 mL/min.

Purged volatiles were analyzed using a Trace Gas Chromatograph Ultra coupled to a Trace ISQ mass spectrometer (Thermo Scientific, Petaluma, CA, USA). Samples were gently heated, causing the volatiles to be dynamically swept along by the helium stream for 30 min at 60 °C. After two minutes of desorption, the volatiles were retained in a trap and subsequently transferred to the chromatograph for analysis. Separation was performed on a Rxi-624Sil MS (30 m × 0.25 mm internal diameter, 1.40 μm film thickness) column from Restek (Bellefonte, PA, USA) using He as the carrier gas at a constant flow rate of 1 mL/min. The temperature program was as follows: initial 35 °C held for 4 min, then raised at 8 °C/min up to 250 °C and held for 5 min. The transfer line and source temperatures were set at 250 °C. The mass spectra were obtained using the electron impact ionization mode at a voltage of 70 eV, and data acquisition was performed in full scan mode over the *m*/*z* range of 20–1000 Da. For data acquisition and processing, Xcalibur 2.0.7 software was used. Compounds were identified using the commercial mass spectral libraries NIST/EPA/NIH 2020 and the Wiley Registry^TM^ 12th edition.

#### 2.4.2. GC-MS

In this case, chromatographic analyses were carried out using a Zebron ZB-5MSi (30 m × 0.25 mm internal diameter, 0.25 µm film thickness) column from Phenomenex^®^ (Torrance, CA, USA) and He as the carrier gas at a constant flow rate of 1 mL/min. The temperature program was as follows: initially 40 °C held for 2 min then raised at 9 °C/min up to 300 °C and held for 10 min. The transfer line and source temperatures were set to 300 °C. Mass spectra were obtained with a mass-selective detector operated under electron impact ionization mode at a voltage of 70 eV and data acquisition was performed in full scan mode over the *m*/*z* range of 35–1000 Da. For data acquisition and processing, Xcalibur 2.0.7 software was used. Compounds were identified using the commercial mass spectral libraries NIST/EPA/NIH 2020 and the Wiley Registry^TM^ 12th edition.

## 3. Results and Discussion

To identify the volatile and semi-volatile compounds in the study samples, as mentioned above, two different extraction techniques were used (P&T for volatiles and extraction with solvents for semi-volatiles), as well as two different GC-MS methods. In both cases, a comparison of the mass spectra obtained for each peak with commercial libraries was made, considering only compounds whose direct matching factors (SI) and reverse search matching (RSI) were greater than 800. In the case that the identification was not possible, the *m*/*z* obtained was indicated. Furthermore, when a commercial standard was available, confirmation was made using the same analytical conditions as those used for the analysis of the samples.

In addition, the known concentration of the IS (internal standard) was used as a reference to estimate the concentration of each compound and thus compare its presence in each sample. Different amounts of IS were tested to obtain chromatographic peaks suitable for this purpose. The selected IS concentration for the analysis of volatile compounds by P&T-GC-MS was found to be 0.1 µg/kg of sample, while for the analysis of semi-volatile compounds, the concentration of IS was 62.5 mg/kg for the analysis of raw materials and 37.5 mg/kg for films.

This approach was used instead of individually quantifying each compound, as the final objective was to monitor whether these compounds increased or decreased throughout or after any change in the manufacturing process.

### 3.1. Volatile Compounds

#### 3.1.1. Screening Analysis of Additives and PE Grades

**Figure 2:**
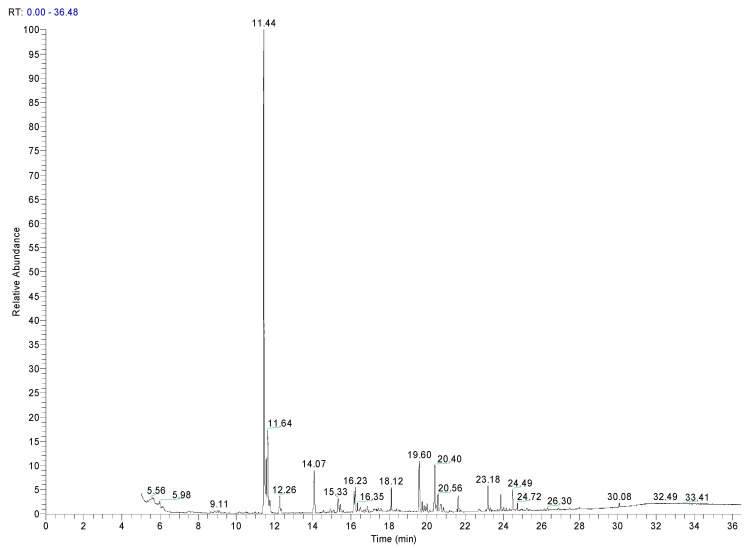
Chromatogram of an additive of PE (Add.3).

Alkanes and alkenes were the most abundant compounds identified in all the samples, and both were PE oligomers from the polymerization residues. Other volatile compounds with benzene structures, such as ethylbenzene or xylenes (m- and p-), were detected in the three additives, although they were not detected in any of the PE samples. Ethylbenzene can be observed as the most intense peak in the chromatogram of Add.3 (Figure 2). This could be due to its use as a solvent. Similarly, toluene was identified in two additives, and both have been identified in other PE materials for food packaging [13].

Aldehydes, such as hexanal, heptanal, or nonanal, were also detected in the PE additives, which can be attributed to the oxidation of polyolefin. Nonanal, a compound commonly described as having fatty, citrus, and green odors, was found in various PP materials [14], and has also been described as a thermal oxidation product in PE [15]. Besides being considered as a degradation product, nonanal can also be used as a solvent [16]. Like aldehydes, ketones are also oxidation products commonly found in polyolefins.

The estimation of the concentration of each compound is shown in Table 4. For this purpose, the area of each peak was compared with that of the IS, which was added at a known concentration, as mentioned above. A summary of the identified compounds, as well as their estimated concentration expressed in µg/kg IS and the deviation expressed in % (each sample was analyzed in duplicate), is shown in Table 4. All peaks with an area below the one obtained for the 0.01 µg IS/kg of sample were not considered.

It is worth highlighting the intensity of peaks at elution times of 6.1 min, 9.9 min, 13.6 min, and 16.8 min in Add.6, corresponding to unknown compounds. Special attention should be paid to these compounds to determine how they could affect the final product throughout the manufacturing process.

In Add.3, the high presence of 2,4-di-tert-butyl phenol (2,4-DTBP) should be noted, which is widely described as a degradation product of the antioxidant Irgafos 168. Additives such as antioxidants or light stabilizers are incorporated into the polymer to enhance their properties. However, they can be degraded into other substances such as 2,4-di-tert-butylphenol or 2,4,6-tritertbutylphenol (the latter also detected in Add.1) when food packaging is exposed to high temperatures. The function of antioxidants is to be oxidized earlier than the material when oxidizing conditions, such as high temperatures, occur. Thus, degradation products of commonly used antioxidants, such as Irganox 1010 and Irgafos 168, have been widely observed [9].

The material that showed the highest quantity of total volatile compounds was Add.6. These compounds were mainly alkanes or unidentified compounds (labeled in Table 4 as unknown compounds). This sample (Add.6, titanium dioxide (TiO_2_)) is added to the PE at up to 7% to protect the polymer from UV radiation and to restrict the permeation of gases [17].

Add.5 was also added to PE at 1% as a processing aid, which can work as an anti-blocking masterbatch agent during the treatment or processing of PE to provide specific technological functions. Production aids are indicated in Regulation 10/2011/EU for plastic materials and articles to come into contact with food as authorized substances used in small amounts in the polymerization so as to not pose any potential risk for human health in the final polymer that will be in contact with food [3].

#### 3.1.2. Optimization of Processing Parameters in the Compounding Stage

Four processing conditions were selected to process the materials in the pilot plant to simulate conditions closer to those used on an industrial scale. The variables considered were the material produced included in the hopper per hour (kg/h), velocity of production (rpm), and applied vacuum. During these tests, the atmospheric vents were left open and only one pump was placed at the end of the extruder (ZSK25 extruder, Coperion GmbH). These applied variables provided eight different reprocessed samples for the PE1 grade, but only three for the PE2 samples for technical reasons (see samples described in Table 2).

All materials were collected and vacuum packed in aluminum bags for subsequent analysis. These previous assays were important for evaluating the effectiveness of the extraction equipment in the reduction of volatile compounds.

In the PE reprocessed samples, alkanes and alkenes were the most abundant compounds detected in all samples, and in most cases were the only identified compounds in these chromatograms, as in the raw materials previously analyzed. However, one difference was observed. Only in samples produced by applying vacuum (PRO_PE1_05, PRO_PE1_06, PRO_PE1_07, PRO_PE1_08), a compound related to a benzene structure attributed to xylene (it was not possible to identify the isomer) with characteristic fragments of *m*/*z* 91 Da and 106 Da was detected.

**Figure 3:**
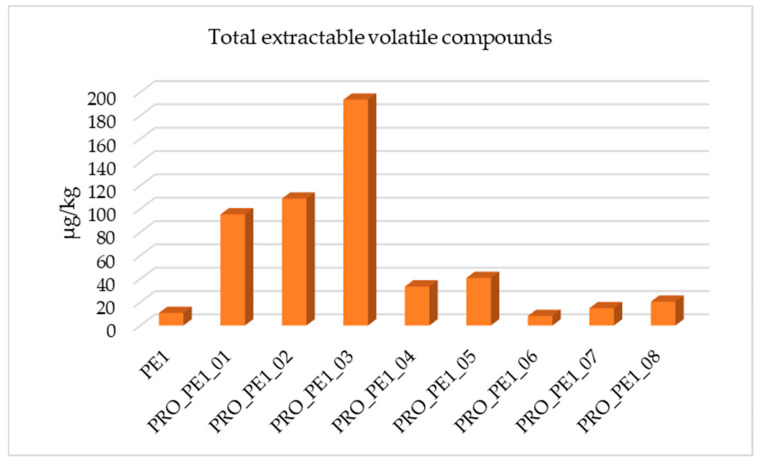
Total extractable volatile compounds identified in PE1 and optimization conditions tested.

All the reprocessed PE samples of PE1 were compared with the original PE sample in terms of the total extractable volatile compounds, as shown in Figure 3. It was observed that those conditions of PRO_PE1_03 showed the highest quantity of detected compounds. This sample corresponded to PE1 processed without vacuum, working at 15 kg and applying 250 rpm. On the other hand, the sample that contained the lowest quantity of volatile compounds was processed by applying vacuum, working at 10 kg and at 400 rpm (PRO_PE1_06).

**Figure 4:**
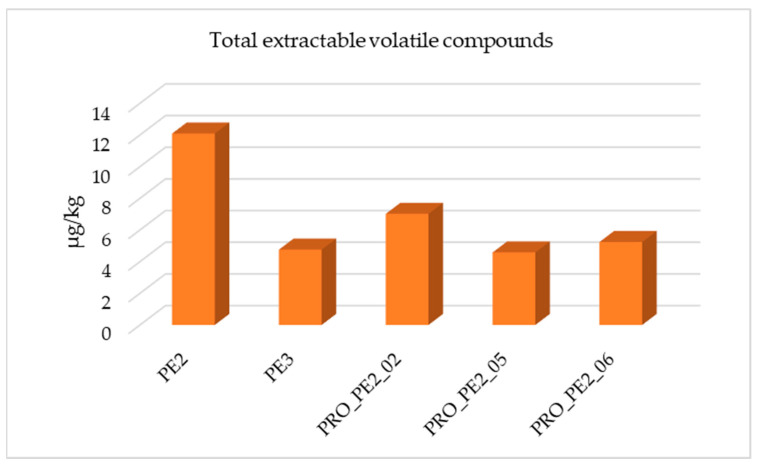
Total extractable volatile compounds identified in PE2 and optimization conditions tested.

The metallocene grades, PE2 and PE3, did not allow testing of the same reprocessing conditions as the aforementioned samples. A similar comparison to that described above is shown in Figure 4, in which PE3 presented the lowest quantity of total volatile organic compounds extracted. This sample was used as the outer layer of the films together with PE1.

PE2 was only processed using 10 kg and 400 rpm with or without vacuum (PRO_PE2_06 and PRO_PE2_02, respectively) and 10 kg and 250 rpm with vacuum (PRO_PE2_05) because of material shortage. The most common compounds detected in all chromatograms were alkanes, alkenes, alcohols, and aldehydes. Additionally, 2,6-di-tert-butyl-p-benzoquinone, which was previously described as a degradation product of the antioxidants Irgafos 168 and Irganox 1010, was detected in PE2 and in the three reprocessed samples (PRO_PE2_02, PRO_PE2_05, and PRO_PE2_06). However, this compound was not found in PE3.

The results for these metallocene grades (PE2 and PE3) were very different to the observed data from PE1, since almost no volatile compounds were extracted using the same chromatographic method. This may be due to the fact that during its processing, the samples caused problems during its cutting.

When processing with different vent ports, the total extractable volatile compounds were reduced, and by applying vacuum, this reduction was much greater (PRO_PE2_05 and PRO_PE2_06).

#### 3.1.3. Analysis of Films

The films were produced by extrusion of the PE grades studied above. Different percentages were used and are summarized in Table 3. All films had three layers and were analyzed following the conditions described in Section 2.2.2 and Section 2.2.3 to identify the volatile compounds present in them (see Table 5).

Alkanes and alkenes were the most abundant compounds identified. Additionally, several alcohols, which either poorly identified or tentatively identified as 1-hexadecanol, were detected in all cases. This compound was present in the processing aids (Add.5 and Add.6) used and was ten times more intense in Add.1. Alcohols (with alkyl chain lengths varying from C-9 to C-16) have been described in the literature as internal lubricants in polymers [16].

Furthermore, other compounds that could be considered NIAS were also identified, such as 2-butanone, which was detected in four of the analyzed films (1, 2, 3, and 6) and was only previously identified in PE3. Another compound observed was methyl 2-methylpropenyl ether, which was detected in three films (1, 2, and 3). However, it was not identified in any of the raw materials. In addition, toluene was detected in seven of the films (1, 2, 3, 4, 6, 8, and 9), which had already been observed in two additives (Add.1 and Add.3) in the analysis of raw materials. 2,4-DTBP was detected again as an impurity in almost all films (1–7); as mentioned above, it was present in three additives (Add.1, Add.3, and Add.4), having the highest concentration in Add.1, which contains the antioxidants Irganox 1010 and Irgafos 168 in a proportion of 1:2. This additive was added during the manufacturing of PE1, which is part of the composition of the three-layers films.

Ethylbenzene (*m*/*z* 91 Da and 106 Da) or one of the isomers previously detected in three additives (Add.1, Add.3, and Add.4), was found in films 1–7.

Concerning the compounds highlighted in the analysis of raw materials, only the compound eluted at 6.1 min (*m*/*z* 75 Da and 105 Da) was present in all the films; another compound, eluted at 9.9 min (*m*/*z* 207 Da), was identified in films 3–8, and two additional peaks, at 13.6 (*m*/*z* 281 Da) and 16.8 min (*m*/*z* 73 Da, 267 Da and 355 Da), respectively, were not detected, although their presence was evident in processing aids (Add.5 and Add.6).

Film 4 was found to contain the highest number of peaks and the most intense ones, followed by films 8 and 9. The films with the lowest number of extractable compounds were films 6 and 7. Thus, once these results were observed, it was concluded that among all non-reprocessed samples, a higher quantity of total volatile extractable compounds was attributed to the presence of aid processing, although this effect was not observed in the reprocessed samples.

### 3.2. Semi-Volatile Compounds

The same procedure and monitoring schemes were used for the identification of semi-volatile compounds from the raw materials to the products obtained after compounding process and film extrusion.

#### 3.2.1. Screening Analysis of Additives and PE Grades

Polar and non-polar solvents were tested to optimize the extraction of additives from PE samples. After extraction with both solvents (ethanol:hexane (3:1), E1, and hexane, E2), the results were compared, and the worst case was chosen to extract the maximum number of compounds in all the samples. According to the available literature [18] and previous experience in the field, E2 extraction proved to be more effective, as hexane has a polarity similar to that of PE, allowing for the extraction of a greater number of compounds. Alkanes and alkenes were the most abundant compounds identified in the PE samples, followed by Irgafos 168, which was present in samples Add.1, Add.2, and Add.3. The oxidation product tris(2,4-di-tert-butylphenyl) phosphate was also observed. Nevertheless, some differences were noted between PE grades.

In PE1, some compounds with an ester structure were detected, although there was not always a good match with the spectral library. Another compound identified was glycerol trycaprylate, which is a lubricant also present in plastic additives [16]. Lubricants are used in this type of material to reduce the adhesion and viscosity of polymers [16].

With respect to the additives employed in the manufacture of PE1 (Add.1 and Add.2), the most intense signals were obtained for compounds identified in their chromatograms as the antioxidants Irgafos 168 (as mentioned above, it was part of Add.1, Add.2 and Add.3) at 36.1 min and Irganox 1010 (contained in Add.2 and Add.3) at 22.4 min. Furthermore, it was present in 2,4-di-tert-butylphenol, which was considered an impurity of the above-mentioned antioxidant Irgafos 168, and an unknown compound with the characteristic ion *m*/*z* 364 Da at 32.15 min, which was not identified.

In PE2 and PE3, the most abundant compound identified was erucamide (Add.4) followed by Irgafos 168 (present in Add.1, Add.2, and Add.3) and its oxidation product. Additionally, in these extracts, 7,9-di-tert-butyl-1-oxaspiro(4,5)deca-6,9-diene-2,8-dione was detected, which has been reported as an NIAS and is considered a degradation product of the antioxidant Irganox 1010. This substance was found in several samples of both plastic and paper packaging in other studies [19].

In the production of PE2 and PE3, two additives (Add.3 and Add.4) were involved. The extraction of Add.3 revealed that the most abundant compounds identified were Irgafos 168 and Irganox 1010. Both compounds serve as long-term stabilizers to protect plastics from thermo-oxidative degradation. Irganox 1010 is also described as an antioxidant of low volatility, odorless, and tasteless, which is suitable for polyolefins, PE, PP, polyamides, polyurethanes, polyesters, and PVC applications [20].

Furthermore, in this additive (Add.3), 2,4-di-tert-butylphenol was also identified, which has been previously detected as a degradation product of Irgafos 168; 2,4,6-tri-tert-butylphenol, which has been reported as a chemical intermediate in the production of antioxidants utilized in the rubber and plastic industry [21]; as well as 3,5-di-tert-butyl-4-hydroxybenzaldehyde and 3,5-di-tert-butyl-4-hydroxyphenylpropionic acid, which are described as degradation products of antioxidants in other works concerning PE [8,9,22]. Additionally, several other compounds whose identification was not satisfactory were also detected at 26.6 min (*m*/*z*: 147, 203, 189, and 219 Da) and at 32.15 min (*m*/*z* 364 Da).

The main compound of the other additive involved in the production of PE2 and PE3 was erucamide (Add.4), which is a common slip agent used in PE to bloom to the surface once the film has been produced, thereby reducing friction coefficients in post-processing operations [23]. Related to compounds belonging to the amide family, 9-octadecenamide (CAS, 3322-62-1) was also tentatively identified and described as a degradation product of hindered amine light stabilizers, such as Chimassorb 944 [24] and oleamide (CAS, 301-02-0). The identification of this erucamide-related compound was not definitive, as both substances exhibit identical characteristic mass fragments (*m*/*z* 59 and 72 Da). Nevertheless, this tentatively identified compound was not intentionally added to these materials.

Furthermore, and maybe related to these compounds, an oleonitrile compound was identified, (Z)-9-octadecene nitrile (CAS, 112-91-4), which is produced when oleic acid is treated with an excess of ammonia [25].

The previously mentioned unknown compound in the Add.3 sample, with an *m*/*z* of 364 Da, was also detected, along with 3,5-di-tert-butyl-4-hydroxyphenylpropionic acid and 3,5-di-t-butyl-4-hydroxybenzaldehyde. The latter compound has also been reported as a degradation product of antioxidants such as octadecyl 3-(3,5-di-tert-butyl-4-hydroxyphenyl) propionate (Irganox 1076, CAS, 2082-79-3) and pentaerythritol tetrakis (3-(3,5-di-tert-butyl-4-hydroxyphenyl) propionate (Irganox 1010, CAS, 6683-19-8), both of which are plastic additives present in polyolefin-based materials [26], although only the second antioxidant was used in these samples. Alkanes, alkenes, and alcohols were also detected, which may be attributed to residuals from the polymerization process.

In relation to the extractions of Add.5 and Add.6, the most abundant compounds identified were alkanes and alkenes, such as those obtained by P&T-GC-MS.

After examining the results obtained from these raw materials, which could represent the initial critical point in NIAS production, it was concluded that the main substances identified were the Intentionally Added Substances (IASs) used in its production, such as antioxidants and slip agents. Additionally, alkanes, alkenes, and oxidation products (aldehydes and ketones) were also observed, which likely resulted from the polymerization process, as this is the stage where polymerization occurred.

#### 3.2.2. Optimization of Processing Parameters in the Compounding Stage

NIAS can also be found in the subsequent additivation stage, known as the compounding process. Samples processed under eight different conditions for PE1 and three different conditions for PE2, detailed in Table 2, were also extracted for the identification of semi-volatile compounds.

**Figure 5:**
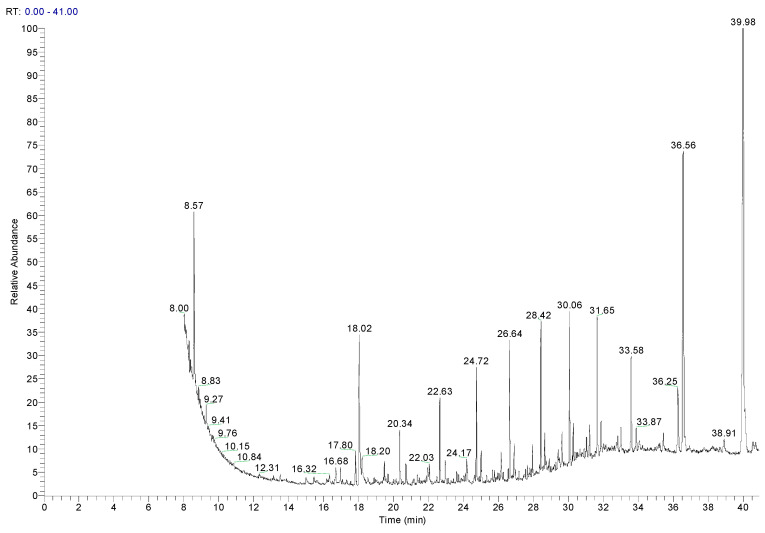
Chromatogram of sample PRO_PE1_07 analyzed by GC-MS after extraction with E2.

The samples were compared with each other, considering the alkanes profile. For PE1, the same substances were identified across all samples: tris(2,4-di-tert-butylphenyl) phosphite (Irgafos 168), 2,4-di-tert-butyl phenol (2,4-DTBP), and tris(2,4-di-tert-butylphenyl) phosphate (oxidized Irgafos 168). The latter two compounds are degradation products of the antioxidant already mentioned and identified before, Irgafos 168 [16]. In all cases, the intensity of the oxidation product of the antioxidant Irgafos 168 was higher than that of the antioxidant itself, indicating that it fulfilled its function. This can be observed in Figure 5, which shows a chromatogram of the processed PE1 sample (PRO_PE1_07), where the two more intense peaks were attributed to the antioxidant used and its oxidation product.

In the case of PE2, only three different conditions were tested, as previously explained. The same antioxidants and degradation products were observed, as well as the same amides cited earlier (known plastic additives used as slip agents), although their presence was lower than in the original PE2. Two other undesired compounds were also identified, which were mentioned in the extraction of Add.3 and Add.4: 7,9-di-tert-butyl-1-oxaspiro(4,5)deca-6,9-diene-2,8-dione and (Z)-9-octadecene nitrile, respectively, both present in lesser quantities in the three processed samples with different processing parameters (PRO_PE2_02, PRO_PE2_05 and PRO_PE2_06). Due to its fluidity and the difficulties in working with this material in the industry, PE3 was used instead of PE2 to produce the films together with PE1, according to the results obtained also in the P&T screenings. In brief, no additional compounds were detected using the solvent extraction screening.

#### 3.2.3. Analysis of Films

The extrusion process during film fabrication can represent another critical point in NIAS generation; therefore, these final products were also studied because they are intended to be materials in contact with food. The films were extracted in one-side migration cells using two pieces of film in each assay, considering only the side in contact with food and the E2 conditions described in Section 2.2.2. Since E2 conditions were found to be more effective in extracting a greater number of compounds from the raw materials, these conditions were selected for application to the films. As in previous results, alkanes and alkenes were the most abundant compounds in all films. Erucamide was also identified, used as a slip agent, and was previously identified in Add.4. The antioxidant Irgafos 168 and its oxidation product (tris(2,4-di-tert-butylphenyl) phosphate) were also observed, as well as other compounds, as shown in Table 6, which were compared with the quantities present in the raw material used to prepare the films.

Thus, these compounds were monitored in detail to obtain more information about the material they came from or at which point of production the contamination was produced. For example, 7,9-di-tert-butyl-1-oxaspiro(4,5)deca-6,9-diene-2,8-dione was identified in all the film extracts and in the PE2 and PE3 samples. The presence of three other substances was observed, although only in one of the replicates of film 8: octanoic acid, nonanoic acid, and another compound that was tentatively identified as 5-dodecylhydro-2(3H)-furanone. As will be shown later, none of them were detected in the previous tests or in the migration tests detailed in Section 3.3.

Glycerol tricaprylate was identified only in film 8, although it was detected in PE1, which was involved in the production of all three-layer films. Film 8 exhibited the highest quantity of semi-volatile extracted and, thus was selected to simulate the worst possible scenario from a safety point of view. Migration assays were performed in an attempt to obtain a more comprehensive study, as described in Section 3.3. In contrast, film 3 presented the lowest amount of total extractable compounds in comparison to all the films analyzed. In this film, all three layers were processed under vacuum and using only 1% Add.5, which was one of the raw materials with the lowest quantity of migratable compounds detected.

### 3.3. Migration Tests

Among all the films analyzed in this work, the worst-case scenario was exhaustively evaluated and selected for the migration tests (film 8). Migration assays were conducted using different food simulants (A, B, and D2 substitute) to cover the widest range of food that could be in contact with these films, as outlined in Regulation 10/2011/EU [3].

Most compounds were detected after migration assays with ethanol 95% (*v*/*v*) (D2 substitute) as a simulant, followed by the migration test using ethanol 10% (*v*/*v*) (A). However, no compounds were detected when acetic acid 3% (*v*/*v*) (B) was utilized. Additionally, these extracts were redissolved in hexane and methanol, obtaining the same result. The concentration of IS used for the migration tests was 0.040 g/kg for simulant A (ethanol 10% *v*/*v*) and 0.075 g/kg for simulant D2 substitute (ethanol 95% *v*/*v*). The results are presented in Table 7.

Several undesired compounds, such as 5-dodecylhydro-2-(3H)-furanone or glycerol trycaprylate, which were previously identified in the extracts of the films, were not detected in migration assays in any of the simulants analyzed. However, 7,9-di-tert-butyl-1-oxaspiro(4,5)deca-6,9-diene-2,8-dione, a degradation product of the antioxidant Irganox 1010, was present in both ethanol 10% *v*/*v* and 95% *v*/*v* simulants.

Irgafos 168, along with its oxidation product and methyl 3-(3,5-di-tert-butyl-4-hydroxyphenyl) propanoate, were identified, although they were present only in the migration assays using ethanol 95% (*v*/*v*). Moreover, for the first time, a compound with a phenolic structure was detected and tentatively identified as 2-tert-butyl-6-[(3-tert-butyl-2-hydroxy-5-methylphenyl)methyl]-4-methylphenol. This could be the “unknown compound” that eluted at 30.0 min (see Table 4) in the results obtained by P&T, as it was also detected in additives Add.1 and Add.3 due to similar *m*/*z* values.

In addition to these compounds, two esters were identified in both migration extracts: methyl hexadecanoate and methyl octadecanoate, which were not detected in previous extracts. Both compounds are typical oxidation products from conventional polyolefins as PE. Some researchers have detected them in polystyrene packaging, as they can be used as polymeric lubricants to reduce friction with the thermoforming equipment and increase its flexibility [5].

Finally, only a small percentage of the compounds identified in extraction assays migrated to the food simulants tested. Specifically, less than 0.1% of total extractable compounds migrated to ethanol 10% (*v*/*v*) and nearly ten more times to ethanol 95% *v*/*v*. These findings align with other studies and current regulations, for example, regarding the migration of phthalates, as all concentrations found in distilled water in contact with PE were below the specific migration limit (SML) [27]. Nevertheless, unidentified substances may still be present and susceptible to migration into the food [28,29,30,31].

Among the substances identified in migration assays, two were confirmed with analytical standards: methyl hexadecanoate and Irgafos 168. Standard solutions of 10 mg/L were prepared and analyzed in the same chromatographic conditions as migration assays. For both compounds, the retention time and the MS spectra of the analytical standards matched the corresponding peaks identified in the migration extracts of film 8.

As previously mentioned, most identified substances were polymer additives, such as plasticizers, antioxidants, or slip agents. Moreover, linear and branched alkanes with the general formula C_n_H_2n+2_, representing low molecular weight polyethylene oligomers, were detected in all PE samples. Many of them can be present as contaminants; however, their presence could also result from the partial degradation of PE or they could be generated during solvent extraction.

In this case, the identification was not possible, although efforts were made. The branched alkanes detected were tentatively identified by calculating the Kovats index. For the retention index (RI), a standard mixture of C7-C30 alkanes was analyzed under the same chromatographic conditions. Consequently, the alkanes detected at 24.6 and 26.5 min were tentatively identified as 3-methylheneicosane (CAS, 6418-47-9) and 11-methyltricosane (CAS, 27538-41-8), respectively, with calculated RIs of 2170 and 2339, and RIs obtained in the library of 2182 and 2333, respectively. No similar matches were obtained for the compound identified as alkane at 28.3 min with a calculated RI of 2550.

Additionally, other substances identified in previous assays but not in the migration tests were confirmed using analytical standards, including toluene, 2,6-di-tert-butyl-p-benzoquinone, 2,4-di-tert-butylphenol, erucamide, and several aldehydes such as hexanal and α-pinene.

## 4. Conclusions

More than 50 volatile compounds were detected by P&T-GC-MS, demonstrating its effectiveness as a screening technique for identifying substances in PE materials. Similar results were obtained in the solvent extraction assays for this material, as only one previously unidentified compound was detected in the migration tests.

The expected results were observed, with alkanes and alkenes being the most abundant compounds detected in all samples (raw materials, reprocessed samples, and films) analyzed in this study. Polyethylene oligomers, decomposition products, and polymerization residues were also detected. The presence of aldehydes was unsurprising because they are oxidation products of polyolefins.

Undesired compounds were also identified using spectral libraries to identify the volatile and semi-volatile compounds. When possible, commercial standards were used to confirm the proposed identification. Some of them are well-known NIAS, such as 2,6-di-tert-butyl-p-benzoquinone or 2,4,6-tri-tert-butylphenol. Moreover, nine substances were successfully confirmed with commercial standards, including 2,4-di-tert-butylphenol, which is described in the literature as a degradation product of Irgafos 168. The methodology applied to these PE samples, intended for use as FCMs, showed the presence of polyethylene oligomers, antioxidants, and lubricants. Furthermore, some compounds remain unidentified, and their presence should not be underestimated. Thus, the *m*/*z* obtained provides useful information for future research in the field of FCM.

All these substances must be considered, and their concentration in food must not pose a hazard to human health. In Regulation 10/2011/EU, which applies to plastic materials intended for food contact, only Irgafos 168 and 2-tert-butyl-6-[(3-tert-butyl-2-hydroxy-5-methylphenyl)methyl]-4-methylphenol are authorized as additives or polymer production aids. Furthermore, in addition to the substances included in the European Union list of authorized substances, this regulation emphasizes that NIAS must comply with the general safety requirements for food-contact materials. Accordingly, the generation of NIAS should be evaluated throughout the production chain.

This study has demonstrated how modifications at the different stages of the film manufacturing process can reduce the presence of NIAS. It has been shown that applying vacuum during the compounding stage and film production reduces the presence of undesirable volatile compounds. Other processing conditions, such as rpm, were material-dependent. Therefore, specific material recommendations for processing conditions to minimize NIAS would be valuable tools that could positively impact consumer safety.

It is worth noting that companies should improve their production processes by taking into account NIAS generation, and also the cooperation among them, considering all critical points during the transformation chain of these materials. This work provides an example of how this important issue could be addressed.

## Figures and Tables

**Table 1:** Samples including PE grade and additive description.

Sample	Formula	Description
PE1	LDPE grade	Pellets
PE2	LDPE grade	Pellets
PE3	LDPE grade	Pellets
Add.1	1:2 Pentaerythritol tetrakis(3-(3,5-di-tert-butyl-4-hydroxyphenyl)propionate) (Irganox 1010, I-1010, CAS 6683-19-8): Tris(2,4-di-tert-butylphenyl) phosphite (Irgafos 168, I-168, CAS 31570-04-4)	White Powder (Additive for PE1)
Add.2	PE + Tris(2,4-di-tert-butylphenyl) phosphite + Pentaerythritol tetrakis(3-(3,5-di-tert-butyl-4-hydroxyphenyl)propionate)	White Pellets (Additive for PE1)
Add.3	Pentaerythritol tetrakis(3-(3,5-di-tert-butyl-4-hydroxyphenyl)propionate) + Tris(2,4-di-tert-butylphenyl) phosphite+ Dynamar FX 5920A (CAS 190454-49-0): Polyethylene glycol (CAS 25322-68-3) + vinylidene fluoridehexafluoropropylene polymer (CAS 9011-17-0) + Calcium carbonate (CAS 471-34-1) + Talc (CAS 14807-96-6)	White cylinders (Additive for PE2)
Add.4	Talc 2 + (Z)-docos-13-enamide (erucamide, CAS 112-84-5) + Zinc oxide (CAS 1314-13-2)	Gray cylinders (Additive for PE2)
Add.5	Processing aid of PE (1%): silica	Pellets (antiblocking masterbatch)
Add.6	White concentrate (≤7%): TiO_2_	Pellets (white masterbatch)

**Table 2:** Pellet samples obtained after processing conditions.

Sample	Description	Production (Kg/h)	Speed (rpm)	Vacuum
PRO_PE1_01	LDPE (PE1)	10	250	no
PRO_PE1_02	LDPE (PE1)	10	400	no
PRO_PE1_03	LDPE (PE1)	15	250	no
PRO_PE1_04	LDPE (PE1)	15	400	no
PRO_PE1_05	LDPE (PE1)	10	250	yes
PRO_PE1_06	LDPE (PE1)	10	400	yes
PRO_PE1_07	LDPE (PE1)	15	250	yes
PRO_PE1_08	LDPE (PE1)	15	400	yes
PRO_PE2_02	Metallocene (PE2)	10	400	no
PRO_PE2_05	Metallocene (PE2)	10	250	yes
PRO_PE2_06	Metallocene (PE2)	10	400	yes

**Table 3:** Film description: composition of the three layers and the different manufacturing processes applied.

Sample	OUT LAYER 1		CORE LAYER 2		OUT LAYER 3		Appearance	Processing Conditions
	Thickness (µm)	Composition (%)	Thickness (µm)	Composition (%)	Thickness (µm)	Composition (%)		
Film 1	12.5	79.5% PE3	25	100% PE1	12.5	79.5% PE3	Transparent	Vacuum not applied
		19.5% PE1				19.5% PE1		
		1% Add.5				1% Add.5		
Film 2	12.5	79.5% PE3	25	100% PE1	12.5	79.5% PE3	Transparent	Vacuum applied in out layers
		19.5% PE1				19.5% PE1		
		1% Add.5				1% Add.5		
Film 3	12.5	79.5% PE3	25	100% PE1	12.5	79.5% PE3	Transparent	Vacuum applied
		19.5% PE1				19.5% PE1		
		1% Add.5				1% Add.5		
Film 4	12.5	79.5% PE3	25	80% PE1	12.5	79.5% PE3	Opaque White	Vacuum not applied
		19.5% PE1				19.5% PE1		
		1% Add.5		20% Add.6		1% Add.5		
Film 5	12.5	79.5% PE3	25	80% PE1	12.5	79.5% PE3	Opaque White	Vacuum applied
		19.5% PE1				19.5% PE1		
		1% Add.5		20% Add.6		1% Add.5		
Film 6	12.5	79.5% PE3	25	100% PE1	12.5	79.5% PE3	Transparent	Vacuum not applied (Production 325 kg/h)
		19.5% PE1				19.5% PE1		
		1% Add.5				1% Add.5		
Film 7	12.5	79.5% PE3	25	100% PE1	12.5	79.5% PE3	Transparent	Vacuum not applied (Production 400 kg/h)
		19.5% PE1				19.5% PE1		
		1% Add.5				1% Add.5		
Film 8	12.5	79.5% PE3	25	80% PE1	12.5	79.5% PE3	Opaque White	Vacuum not applied (Production 325 kg/h)
		19.5% PE1				19.5% PE1		
		1% Add.5		20% Add.6		1% Add.5		
Film 9	12.5	79.5% PE3	25	80% PE1	12.5	79.5% PE3	Opaque White	Vacuum not applied (Production 400 kg/h)
		19.5% PE1				19.5% PE1		
		1% Add.5		20% Add.6		1% Add.5		

**Table 4:** Compounds detected in the additives for PE, concentration relative to the IS and standard deviation (SD) in %.

Rt/min	Compound	CAS	Add.1	Add.2	Add.3	Add.4	Add.5	Add.6
			µg/kg (SD)	µg/kg (SD)	µg/kg (SD)	µg/kg (SD)	µg/kg (SD)	µg/kg (SD)
5.2	2-Ethyl-2-methyloxirane	30095-63-7	-	-	-	0.04 (0.01)	0.04 (0.04)	0.09
6.1	Dimethoxydimethylsilane	1112-39-6	2.15 (0.61)	1.02 (0.7)	-	-	0.65 (0.47)	14.79 (1.5)
7.1	Heptane	142-82-5	-	-	-	0.05 (0.01)	0.03 (0.005)	-
8.7	Acetal structure (*m*/*z* 55, 75, 85)		-	-	0.12 (0.1)	-	-	-
8.8	2,2-Dimethoxybutane	3453-99-4	-	-	0.11 (0.09)	0.06	0.01	0.42
9.1	Toluene	108-88-3	0.17 (0.03)	-	0.09 (0.04)	-	0.02 (0.004)	-
9.4	Octane	111-65-9	-	-	-	0.06 (0.02)	-	-
9.9	Unknown compound (*m*/*z* 207)		0.17 (0.05)	-	-	-	0.08 (0.04)	37.15 (51.75)
10.1	Hexanal	66-25-1	-	-	0.11 (0.08)	0.6 (0.29)	0.03 (0.0003)	-
10.4	Ethylcyclopentane	1640-89-7	-	-	0.02 (0.01)	-	-	-
10.5	Ethylcyclohexane	1678-91-7	-	-	0.07 (0.04)	-	-	0.34
10.6	1,1,3-Trimethylcyclohexane	3073-66-3	-	-	0.04 (0.03)	-	-	-
11	4-Methyloctane	2216-34-4	-	-	-	-	0.01 (0.002)	-
11.4	Ethylbenzene	100-41-4	0.17 (0.11)	-	22.1 (22.68)	0.07 (0.05)	0.01	-
11.8	Nonane	111-84-2	-	-	-	0.01	0.01 (0.002)	-
12.3	p, m, o-Xylene		0.06 (0.01)	-	0.85 (0.77)	-	-	0.39
12.5	Heptanal	111-71-7	-	-	-	0.12 (0.02)	-	-
12.84	Methyl hexanoate	106-70-7	-	-	-	0.04 (0.01)	-	-
13	α-Pinene	80-56-8	-	-	-	0.02 (0.01)	0.02 (0.004)	0.02
13.6	Unknown compound (*m*/*z* 281)		-	-	-	-	0.12 (0.02)	15.59 (7.6)
14.1	Alkane		2.58 (0.6)	1.93 (1.36)	2.18 (1.99)	0.25 (0.005)	0.49 (0.06)	0.59 (0.06)
14.6	Benzene structure (*m*/*z* 105, 120)		-	-	-	-	0	-
15.1	Cyclohexene strcucture (*m*/*z* 68, 93)		0.19 (0.03)	0.04 (0.02)	-	-	0.02 (0.001)	-
15.3–15.5	Alkane		0.29 (0.24)	0.15 (0.12)	0.93 (0.82)	0.42 (0.02)	0.29 (0.04)	1.15 (0.94)
16.2–16.4	Alkane		1.06 (0.23)	0.2 (0.15)	0.23 (0.33)	0.63 (0.27)	0.52 (0.06)	0.74 (0.39)
16.8	Unknown compound (*m*/*z* 267, 355)		-	-	-	-	0.09 (0.04)	13.06 (6.53)
16.9	Nonanal	124-19-6	0.38 (0.27)	0.02 (0.02)	0.2 (0.19)	0.19 (0.01)	-	-
17.6	Alkane		0.43 (0.07)	0.01	0.01	-	0.03 (0.01)	0.07 (0.05)
18.1	Alkane		1.67 (0.38)	0.15 (0.1)	0.64 (0.73)	0.05	1.01 (0.09)	1.6 (1.36)
18.6	2-Decanone	693-54-9	0.15 (0.02)	0.01 (0.01)	-	0.05	-	-
19.6–20.0	Alkane		8.09 (1.51)	0.39 (0.44)	2.56 (3.02)	1.56 (0.07)	2.39 (0.9)	17.58 (3.41)
20.4–20.9	Alkane		8.63 (1.16)	0.06 (0.05)	2.84 (3.39)	1.37 (0.02)	0.91 (0.03)	0.52
21.6	Alkane		2.13 (0.54)	0.02 (0.01)	0.41 (0.49)	0.11 (0.13)	0.48 (0.23)	0.79 (0.43)
23.9	Alkane		3.69 (1.11)	-	0.46 (0.57)	0.13 (0.01)	-	-
24.5	2,4-Di-tert-butylphenol (2,4-DTBP)		4.04 (0.53)	-	0.62 (0.67)	0.03	0.21 (0.04)	-
25.2	2,4,6-Tri-tert-butylphenol	732-26-3	1.26 (0.55)	-	0.09 (0.11)	-	-	-
25.5	Ester structure		-	-	-	-	0.09 (0.04)	-
28	Benzenediol structure		-	-	0.01	0.03 (0.01)	-	-
30	Unknown compound (*m*/*z* 57, 147, 277)		4.76 (2.19)	-	0.11 (0.12)	-	-	-
	SUM		49.7 (10.2)	4.3 (3.1)	35.9 (36.9)	6.4 (1.2)	9.1 (1.2)	109.8 (70.5)

**Table 5:** Compounds identified in samples 1-9 and PE grades used in their manufacture by P&T-GC-MS.

Rt/ min	Compound	CAS	Film 1	Film 2	Film 3	Film 4	Film 5	Film 6	Film 7	Film 8	Film 9	PE1	PE3
			µg/kg (SD)	µg/kg (SD)	µg/kg (SD)	µg/kg (SD)	µg/kg (SD)	µg/kg (SD)	µg/kg (SD)	µg/kg (SD)	µg/kg (SD)	µg/kg (SD)	µg/kg (SD)
5.3	2-Butanone	78-93-3	0.01 (0.001)	0.02 (0.0005)	0.02 (0.0003)	-	-	0.02 (0.001)	0.04	-	-	-	-
6	Methyl 2-methylpropenyl ether	17574-84-4	0.01 (0.002)	0.01	0.01 (0.003)	-	-	-	0.01	-	-	-	-
6.1	Unknown compound (*m*/*z* 75, 105)		0.12 (0.13)	0.05 (0.01)	0.07 (0.04)	6.08 (0.77)	1.17 (0.74)	0.19 (0.13)	0.05 (0.01)	3.62 (2.6)	3.8 (2.07)	-	-
9.1	Toluene	108-88-3	0.02 (0.003)	0.1 (0.11)	0.01 (0.004)	0.01	0.01	0.01 (0.001)	0.02	0.02 (0.004)	0.03 (0.003)	-	-
9.9	Unknown compound (*m*/*z* 207)		-	-	0.02	6.08	1.8	0.02	0.02	3.5	3.56	-	-
11.7	Benzene structure		0.03 (0.0001)	0.04 (0.03)	0.03 (0.004)	0.07 (0.04)	0.02 (0.004)	0.01 (0.001)	0.01 (0.001)	-	-	-	-
22.7	1-Hexadecanol	36653-82-4	0.1 (0.04)	0.16 (0.01)	0.1 (0.02)	0.29 (0.36)	0.28 (0.16)	0.24 (0.002)	0.15 (0.08)	0.54 (0.25)	0.47	0.13	-
24.5	2,4-DTBP	96-76-4	0.02 (0.004)	0.04 (0.02)	0.05	0.02	0.28 (0.34)	0.03	0.12	0.35	0.54	-	0.01 (0.002)
	Sum (with alkanes and alkenes)		72.7 (3.6)	93 (25.3)	55.4 (6.9)	190.1 (6.9)	68.7 (7.9)	40.7 (8.9)	46.7 (13.5)	107.6 (32.4)	140.6 (14.1)	11.7 (0.82)	5.57 (1.15)

**Table 6:** Compounds identified in the extraction of samples 1 to 9 and PE grades by GC-MS.

Rt/min	Compound	CAS	Film 1	Film 2	Film 3	Film 4	Film 5	Film 6	Film 7	Film 8	Film 9	PE1	PE3
			mg/kg (SD)	mg/kg (SD)	mg/kg (SD)	mg/kg (SD)	mg/kg (SD)	mg/kg (SD)	mg/kg (SD)	mg/kg (SD)	mg/kg (SD)	mg/kg (SD)	mg/kg (SD)
11.97	Octanoic acid	57-11-4	-	-	-	-	-	-	-	31.68	-	-	-
13.54	Nonanoic acid	646-30-0	-	-	-	-	-	-	-	50.5	-	-	-
21.8	7,9-Di-tert-butyl-1-oxaspiro(4,5)deca-6,9-diene-2,8-dione	82304-66-3	8.47 (3.87)	8.41 (6.18)	6.96 (1.34)	7.25 (2.2)	7.29 (1.53)	3.43 (0.71)	6.23 (2.96)	7.13 (1.46)	8.43 (2.04)	-	6.83 (4.02)
23.93	2(3H)-Furanone, 5-dodecylhydro-	730-46-1	-	-	-	-	-	-	-	7.5	-	-	-
24.54	Decanedioic acid, dibutyl ester	109-43-3	-	-	-	-	-	80.66 (99.6)	6.24	25.13 (20.01)	-	-	-
30.03	Erucamide	112-84-5	65.47 (26.08)	65.27 (61.22)	81.94 (11.07)	75.45 (10.27)	94.08 (1.75)	43.81 (27.81)	57.79 (5.1)	85.25 (20.6)	63.02 (12.72)	-	252.52 (8.99)
31.2	Glycerol tricaprylate	538-23-8	-	-	-	-	-	-	-	66.01 (68.55)	-	26.61	-
36.32	Irgafos 168	31570-04-4	41.77 (13.21)	61.64	-	22.71	8.49	32.87 (22.9)	62.18 (25.86)	23.47 (21.55)	29.55 (3.74)	125.86 (24.46)	200.37 (25.37)
39.74	Irgafos 168 oxidation product	95906-11-9	177.39 (175.66)	144.93 (108.14)	199.85 (42.4)	210.03 (78.11)	242.65 (74.25)	176.88 (70.22)	308.37 (82.53)	302.95 (109.67)	395.17 (124.13)	38.25 (5.1)	96.03 (18.4)
	Sum (with alkanes)		475.2 (240.7)	414.1 (311.8)	426.3 (45.7)	472.4 (89.9)	480.1 (83.3)	484.0 (281.0)	610.6 (159.3)	904.3 (179.5)	658.1 (152.1)	407.6 (27.4)	693.1 (34.4)

**Table 7:** Compounds identified in migration assays using film 8.

Rt/min	Compound	CAS	Structure or *m*/*z*	Ethanol 10% *v*/*v*	Ethanol 95% *v*/*v*
				g/kg(SD)	g/kg (SD)
21.8	7,9-Di-tert-butyl-1-oxaspiro(4,5)deca-6,9-diene-2,8-dione	82304-66-3	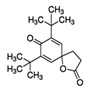	0.004 (0.001)	0.01 (0.01)
21.98	Methyl hexadecanoate	112-39-0	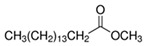	0.01 (0.0002)	0.01 (0.001)
22.07	Methyl 3-(3,5-di-tert-butyl-4-hydroxyphenyl)propanoate	6386-38-5	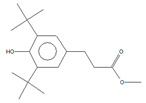	-	0.04 (0.004)
24.12	Methyl octadecanoate	112-61-8	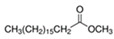	0.01 (0.001)	0.10 (0.02)
24.57	Alkane			-	0.03 (0.02)
26.5	Alkane			-	0.02 (0.001)
26.89	2-Tert-butyl-6-[(3-tert-butyl-2-hydroxy-5-methylphenyl)methyl]-4-methylphenol	119-47-1	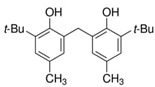	0.04 (0.036)	0.01 (0.003)
28.28	Alkane			-	0.01 (0.001)
36.32	Irgafos 168	31570-04-4	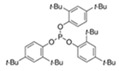	-	1.07 (0.39)
39.74	Irgafos 168 oxidation product	95906-11-9	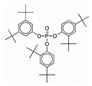	-	0.31 (0.08)
	Sum			0.1 (0.03)	1.6 (0.5)

## Data Availability

The original contributions presented in this study are included in the article. Further inquiries can be directed to the corresponding author.

## References

[1] Groh K.J., Backhaus T., Carney-Almroth B., Geueke B., Inostroza P.A., Lennquist A., Leslie H.A., Maffini M., Slunge D., Trasande L. (2019). Overview of known plastic packaging-associated chemicals and their hazards. Sci. Total Environ..

[2] Gerassimidou S., Geueke B., Groh K.J., Muncke J., Hahladakis J.N., Martin O.V., Iacovidou E. (2023). Unpacking the complexity of the polyethylene food contact articles value chain: A chemicals perspective. J. Hazard. Mater..

[3] COMMISSION REGULATION (EU) No 10/2011 of 14 January 2011 on Plastic Materials and Articles Intended to Come into Contact with Food and Amendments. http://data.europa.eu/eli/reg/2011/10/2023-08-31.

[4] Allahvaisi S. (2012). Polypropylene in the Industry of Food Packaging. Polypropylene.

[5] Galotto M.J., Torres A., Guarda A., Moraga N., Romero J. (2011). Experimental and theoretical study of LDPE versus different concentrations of Irganox 1076 and different thickness. Food Res. Int..

[6] Lahimer M.C., Ayed N., Horriche J., Belgaied S. (2017). Characterization of plastic packaging additives: Food contact, stability and toxicity. Arab. J. Chem..

[7] Alin J., Hakkarainen M. (2011). Microwave Heating Causes Rapid Degradation of Antioxidants in Polypropylene Packaging, Leading to Greatly Increased Specific Migration to Food Simulants as Shown by ESI-MS and GC-MS. J. Agric. Food Chem..

[8] Denberg M., Mosbæk H., Hassager O., Arvin E. (2009). Determination of the concentration profile and homogeneity of antioxidants and degradation products in a cross-linked polyethylene type A (PEXa) pipe. Polym. Test..

[9] Nerin C., Alfaro P., Aznar M., Domeño C. (2013). The challenge of identifying non-intentionally added substances from food packaging materials: A review. Anal. Chim. Acta.

[10] Muncke J., Andersson A.M., Backhaus T., Boucher J.M., Carney Almroth B., Castillo Castillo A., Chevrier J., Demeneix B.A., Emmanuel J.A., Fini J.B. (2020). Impacts of food contact chemicals on human health: A consensus statement. Environ. Health.

[11] Chen Z., Lin Q., Su Q., Zhong H., Nerin C. (2021). Identification of recycled polyethylene and virgin polyethylene based on untargeted migrants. Food Packag. Shelf Life.

[12] De Tandt E., Demuytere C., Van Asbroeck E., Moerman H., Mys N., Vyncke G., Delva L., Vermeulen A., Ragaert P., De Meester S. (2021). A recycler’s perspective on the implications of REACH and food contact material (FCM) regulations for the mechanical recycling of FCM plastics. Waste Manag..

[13] Vera P., Canellas E., Nerín C. (2020). Compounds responsible for off-odors in several samples composed by polypropylene, polyethylene, paper and cardboard used as food packaging materials. Food Chem..

[14] Aldrian O., Czerny M., Buettner A. (2009). Characterisation of flavour compounds formed by gamma-irradiation of polypropylene. Polym. Degrad. Stab..

[15] Wiedmer C., Velasco-Schon C., Buettner A. (2007). Characterization of off-odours and potentially harmful substances in a fancy dress accessory handbag for children. Sci. Rep..

[16] Rani M., Shim W.J., Han G.M., Jang M., Al-Odaini N.A., Song Y.K., Hong S.H. (2015). Qualitative analysis of additives in plastic marine debris and its new products. ArchIves Environ. Contam. Toxicol..

[17] Sangroniz L., Ruiz J.L., Sangroniz A., Fernández M., Etxeberria A., Müller A.J., Santamaria A. (2018). Polyethylene terephthalate/low density polyethylene/titanium dioxide blend nanocomposites: Morphology, crystallinity, rheology, and transport properties. J. Appl. Polym. Sci..

[18] Allen N.S., Sánchez K.T., Edge M., Liauw C.M., Hussain S., Hall K. (2023). Effect of type of polymerization catalyst system on the degradation and stabilization of polyethylenes in the melt state—Part 4: Comparative antioxidant effectiveness on organoleptic extractables. J. Vinyl Addit. Technol..

[19] Graíño S.G., Sendón R., Hernández J.L., de Quirós A.R.-B. (2018). GC-MS Screening Analysis for the Identification of Potential Migrants in Plastic and Paper-Based Candy Wrappers. Polymers.

[20] https://polymer-additives.specialchem.com/product/a-basf-irganox-1010.

[21] OSPAR Commission 2006 Update: OSPAR Background Document on 2,4,6-tri-tert-Butylphenol. ISBN 978-1-905859-01-6. Publication Number: 274/2006. https://www.ospar.org/documents?v=6977.

[22] Burman L., Albertsson A.-C., Höglund A. (2005). Solid-phase microextraction for qualitative and quantitative determination of migrated degradation products of antioxidants in an organic aqueous solution. J. Chromatogr. A.

[23] Agustina L.A., Lestari Y.D., Adhinanda A.A., Ariesta M.N., Choi J., Prananto Y.P., Febriani R. (2024). Study of inorganic based anti-blocks as migration control of slip additive on surface polyethylene monolayer film. Acta Chim. Asiana.

[24] Haider N., Karlsson S. (2001). Loss of Chimassorb 944 from LDPE and Identification of Additive Degradation Products after Exposure to Water, Air and Compost. Polym. Degrad. Stab..

[25] Corma A., Iborra S., Velty A. (2007). Chemical Routes for the Transformation of Biomass into Chemicals. Chem. Rev..

[26] Dorey S., Gaston F., Girard-Perier N., Dupuy N., Marque S.R.A., Barbaroux M., Audran G. (2020). Identification of chemical species created during γ-irradiation of antioxidant used in polyethylene and polyethylene- co -vinyl acetate multilayer film. J. Appl. Polym. Sci..

[27] Ayamba A.A.M., Agyekum A.A., Derick C., Dontoh D. (2020). Assessment of phthalate migration in polyethylene food contact materials sold on the Ghanaian market. Cogent Environ. Sci..

[28] Ong A.H.T., Samsudin H., Soto-Valdez H. (2020). Migration of endocrine-disrupting chemicals into food from plastic packaging materials: An overview of chemical risk assessment, techniques to monitor migration, and international regulations. Crit. Rev. Food Sci. Nutr..

[29] Cropper M., Dunlop S.S., Hinshaw H., Landrigan P., Park Y., Symeonides C. (2024). The benefits of removing toxic chemicals from plastics. Proc. Natl. Acad. Sci. USA.

[30] Tsochatzis E.D., Lopes J.A., Hoekstra E., Emons H. (2020). Development and validation of a multi-analyte GC-MS method for the determination of 84 substances from plastic food contact materials. Anal. Bioanal. Chem..

[31] Schmid P., Welle F. (2020). Chemical Migration from Beverage Packaging Materials—A Review. Beverages.

